# A multivariate analysis of the relationships among the Big Five personality traits, activity-oriented learning styles, and academic performance of Grade 12 students in Thailand

**DOI:** 10.1186/s40359-025-03387-4

**Published:** 2025-09-29

**Authors:** Chayaporn Kaoropthai, Arthur Dryver

**Affiliations:** 1 School of Liberal Arts, Mae Fah Luang University, Chiang Rai, 57100 Thailand; 2 Business School, National Institute of Development Administration, Bangkok, Thailand

**Keywords:** Academic performance, Learning styles, Personality traits, Pre-university students

## Abstract

**Background:**

Research studies show that different personality type students tend to have their own learning styles. Personality traits and learning styles have played a significant role in the academic success of students. However, most of the studies used a more popularized learning styles instrument such as Kolb’s, VARK, or Felder-Silverman’s learning styles, for data collection. This study examined the relationships among the Big Five, learning styles, and academic performance of G12 students.

**Methods:**

A multivariate analysis of variance (MANOVA) statistical technique was chosen to investigate two dependent variables that were continuous (GPA and QPT scores), whereas the independent variables and the confounding variables, gender and school were all categorial. The IPIP Big Five personality markers, the Learning Styles Indicator (LSI) scales, and the Quick Placement Test (QPT) were employed to collect the data. Students’ grade point averages (GPAs) were also used. Purposive sampling was employed, comprising 1,358 students from the three largest government secondary schools in Chiang Rai, Thailand: one school from each of the three districts.

**Results:**

Overall, the results confirmed that there were significant relationships among the Big Five, learning styles, and academic performance, and there was a significant relationship between personality types and learning styles. Agreeableness and conscientiousness were found to be the first two dominant personality types, whereas GAO (group activity orientation) and PO (project orientation) were preferable to IAO (individual activity orientation).

**Conclusion:**

The multivariate results reveal that GPA and QPT were significantly related to school, gender, and learning styles, but were related to personality types non-significantly. The LSI scales have been proved to be practical and appropriate to determine EFL G12 students’ learning styles. The study has contributed to the body of knowledge about personality types and learning styles of G12 students in Thailand. The findings suggest that tailoring teaching methods to students’ learning styles could improve academic performance, especially in English proficiency.

## Introduction

Individual differences, especially personality traits and learning styles, have played an important role in students’ academic success. [12, 19, 30, 32, 39, 47, 57]. Research studies have revealed that different personality type students tend to have their own preferred learning styles [12]. Personality traits denote ones’ characteristic patterns of thoughts, feelings, and behaviors. The most widely used system of traits is the Big Five personality traits, which include five traits (OCEAN): openness, conscientiousness, extraversion, agreeableness, and neuroticism [22]. Learning styles are generally the preferred ways that students learn. Learning styles can be described as a set of factors, behaviors, and attitudes that facilitate learning for students in given situations, and learning styles are investigated using a variety of measures: Kolb’s [28] learning styles, VARK learning styles (Fleming, 1995), Schmeck et al.’s [56] learning styles, Reid’s [53] learning styles, etc. These measures provide different classifications of learning styles based on the factors they focus on.

Kolb’s learning styles [28], as one of the most widely used learning style models, are based on the Experiential Learning Theory. In Kolb’s theory, learners are responsible for guiding their learning process in experiential learning. Kolb’s four-step learning process includes: concrete learning, reflective observation, abstract conceptualization, and active experimentation. The VARK learning styles questionnaire (1995) is used to identify a person’s sensory modality preference in learning. It classifies the learner into four learning modes: visual (V), aural (A), read/write (R), and kinaesthetic (K). Reid’s learning style questionnaire (1987) is an instrument used to identify the perceptual learning style preferences of nonnative speakers of English. Reid classifies learners into four different learning modes: the visual learning mode (reading, study charts), auditor learning (listening to lectures, audiotapes), kinaesthetic learning (experiential learning, that is total physical involvement with a learning situation), and tactile learning (“hands-on” learning, such as building models of doing laboratory experiments). Although extensive research studies involving learning styles have been carried out, most of them have focused on sensory or perceptual modalities. It is thus interesting to investigate the use of different approaches in identifying students’ learning styles in order to accommodate the considerable change in current educational environments.

This study aims to answer whether the Big Five personality traits and activity-oriented learning styles significantly predict GPA and English proficiency among Grade 12 students in Thailand. The Big Five personality traits (Openness, Conscientiousness, Extraversion, Agreeableness, Neuroticism) has been commonly used to assess individual differences in personality. In addition, activity-oriented learning styles, including Group Activity Orientation (GAO), Individual Activity Orientation (IAO), and Project Orientation (PO), is chosen to determine students’ preferred learning styles.

## The Big Five personality traits

The Big Five personality traits or the Five-factor model has been commonly used to reveal individuals’ personality traits [7, 15, 22, 29, 30, 32, 39, 41, 48, 62]. The five basic personality trait theory was initiated by Fiske [10], and further developed by many researchers such as Norman [42], Smith and Kihlstrom [59], Goldberg (1981), and McCrae and Costa (1987). The Big Five personality traits can be considered as five broad personality dimensions or OCEAN, as shown in Table 1.

**Table 1:** Big Five personality traits

Trait	Description
Openness	Characterized by imagination, intellectual curiosity, and creativity [38]
Conscientiousness	Involves being organized, dependable, and goal-oriented (John & Srivastava, 1999)
Extraversion	Defined by sociability, assertiveness, and energy [38]
Agreeableness	Reflects cooperation, kindness, and interpersonal trust (John & Srivastava, 1999)
Neuroticism	Tendency toward anxiety, emotional instability, and mood swings [38]

The instruments widely used for measuring the Big Five personality traits include:

- The 60-item NEO-FFI [37]
- The 44-item Big Five inventory (BFI) [21]
- The 240-item NEO-PI-R [4]
- The 100-item IPIP Scales [13]
- The 50-item IPIP Scales [13]
- The IPIP Big-Five personality markers [14]
- The BFI-10 [50]

Among these prominent instruments, the items and scales from the International Personality Item Pool (IPIP) have been commonly used. According to Goldberg and colleagues (2006), the IPIP items have been translated from English into at least 25 other languages, and a lot of research studies using IPIP scales have been published. The factors contributing to the growing popularity of the IPIP include: “(1) it is cost free; (2) its items can be obtained instantaneously via the Internet; (3) it includes over 2000 items, all easily available for inspection; (4) scoring keys for IPIP scales are provided, and (5) its items can be presented in any order, interspersed with other items, reworded, translated into other languages, and administered on the World Wide Web without asking permission of anyone” [15], pp. 84–85). The IPIP scales have exhibited high internal reliability (alpha = 0.84) and have correlated strongly with the major personality traits assessed by the NEO-FFI and the EPQ-R Short Form [14, 16]. Furthermore, previous research (e.g., [3]) shows that traits like conscientiousness and openness predict academic achievement, setting the stage for this study.

## Learning styles

Learning styles can be defined as “cognitive, affective, and psychological characteristics that are stable indicators of the way learners learn and respond to learning environments” (Felder & Spurlin, 2005; [33, 47]). Learning styles can be referred to as “the concept that individuals differ in regard to what mode of instruction or study is most effective for them” [46], p.108). According to Oxford and Anderson [44], at least 20 style dimensions have been identified, but only eight of them would be particularly relevant to second language learning. Among these 20 style dimensions, perceptual learning styles are most popularly employed in second language learning research [18].

According to Kolb [28], learning comprises two intersecting dimensions: concrete experience and abstract conceptualization, and there are four types of learning styles: diverging, assimilating, converging, and accommodating [2, 19]. VARK learning styles or the VARK model was developed by Fleming and Mills [11]. In this learning style model, the VARK questionnaire is used to measure four learning styles: visual (V), aural (A), reading/writing (R), and kinaesthetic (K). In the VARK model, a learner may possess more than one learning style [40]. Schmeck et al.’s [56] learning styles involve a conceptual framework of effective information processing that identifies learning strategies that are likely to enhance learning and academic performance as it is believed that the quality of thinking and depth of processing will affect learning outcome and memory [5, 30, 32]. Their four learning styles comprise: fact retention, methodical study, elaborative processing, and the synthesis-analysis learning style.

The Felder Silverman learning style model (FSLSM) (1988) consists of four dimensions. The first dimension differentiates an active from a reflective way of processing information. The second dimension concerns sensing versus intuitive learning. The third, the visual-verbal dimension deals with learners who remember well and those who get more out of textual representations. Lastly, the fourth dimension categorizes learners as sequential learners versus global learners [17].

Reid’s [51] Perceptual Learning Style Performance Questionnaire (PLSPQ) classifies the way in which individuals primarily learn into six learning styles: (1) visual learners (with their eyes),(2) auditory learners (with their ears); (3) kinaesthetic learners (by experience); (4) tactile learners (by “hands-on” tasks); (5) the group learning style (those that prefer to work in groups); and (6) the individual learning style (those that enjoy working alone). The PLSPQ has been commonly used in L2 research to investigate ESL/EFL students’ learning styles [6, 18, 54, 60, 63]. Although this instrument has gained popularity in its use, “difficulties arise when researchers attempt to conceptualize actual learning styles and relate these to factors other than individual differences” [63], p. 385). Previous research also has reported concerns with the reliability and validity of the instrument [6, 20, 52].

In response to the concerns and difficulties mentioned, the reliability and validity of the PLSPQ were further investigated. Wintergerst and colleagues [63] examined the reliability and validity of Reid’s PLSPQ using exploratory factor analysis to explore its dimensionality. The results revealed that “specific survey items did not necessarily group into factors conceptually compatible with Reid’s learning style model” (p.385). Based on conceptual considerations and new underlying factor structure results, an alternative learning style model, namely “The Learning Style Indicator (LSI),” was proposed. The three-factor solution results using loadings of 0.35 or greater are clustered as follows: Factor 1 consisting of 11 items that focus on group activities or interaction, (Group Activity Orientation); Factor 2 consisting of six items involving individual learning activities (Individual Activity Orientation); and Factor 3 containing seven items concerning participating in a class project (Project Orientation). The LSI was found to have high internal reliability (Cronbach’s alpha = 0.85).

## Literature review

A meta-analysis of 40-year research studies on “the Big Five personality traits and L2 learning achievement” has been recently conducted by Chen and colleagues [3]. The study covered “137 correlation coefficients from 31 primary studies conducted in 24 countries, with a total cumulative sample size of 8853 and published between 1982 and 2020” (p. 851). The findings indicated that L2 students’ academic performance had a significant positive relationship with openness, conscientiousness, extraversion, and agreeableness, whereas its relationship with neuroticism was non-significantly negative. Furthermore, other variables such as age, school regions, and schooling levels also accounted for variations in the correlations between the Big Five and L2 students’ academic performance. In another meta-analysis of the relationships between the Big Five and academic performance, Mammadov [35] found that apart from academic ability, conscientiousness accounted for 28% of the variance in academic performance. Moreover, the relationship of academic performance with openness, extraversion, and agreeableness revealed significantly larger effect sizes at the lower school levels than at the upper levels.

## The Big Five and academic success

Several research studies have investigated the relationship between students’ personality traits and academic performance. Komarraju and colleagues [29] for example examined the role of the Big Five personality traits in predicting college students’ academic success. The results revealed significant relationships among personality traits, motivation, and academic achievement. It was found that the four personality traits (conscientiousness, openness, neuroticism, and agreeableness) accounted for 14% of the variance in GPA. A study entitled “Big Five personality traits and academic performance in Russian Universities” [43], using machine learning, indicated that introversion, agreeableness, neuroticism, and openness had significant relationships with academic achievement, whereas conscientiousness was found to be relatively less important. Further, Meyer and colleagues [39] investigated the relationship between the personality traits and academic achievement of 3,637 upper-secondary school students in Germany and the results revealed that conscientiousness predicted grades and final exams in both mathematics and English. Additionally, conscientiousness had a significant relationship with mathematic test scores, whereas openness was positively related to English test scores. Another research study [41] examined the relationship between the Big Five personality traits and the academic achievement of 2,145 senior high school students in Indonesia, and the findings indicated that all five personality traits were significant predictors of students’ academic performance. Interestingly, the two most significant predictors appeared to be emotional stability and openness to experience.

## Learning styles and academic success

Quite a few researchers have been interested in investigating the role of learning styles in students’ academic achievement. Bailey et al. [1] used the Productivity Environmental Preference Survey [8] as a learning style instrument to examine the relationship between learning styles and the foreign language achievement of 100 university students enrolled in either French or Spanish. The results revealed that higher achievers tended “to like informal classroom designs and to prefer not to receive information via the kinaesthetic mode,” and variables such as responsibility and mobility could enhance “the predictive power of classroom design preference and kinaesthetic orientation with respect to achievement.”

Recently, research studies on the relationship between learning styles and academic success have been conducted with university students in areas such as healthcare, medical sciences, and English. Kamal and colleagues [23] for example evaluated the learning styles of 137 healthcare students using the VARK learning styles questionnaire [28]. The results indicated that 119 participants (86.85) were unimodal, whereas the rest 18 (13.2%) were Multimodal. Among the unimodal learning style, visual (32%) and read/write (26%) were the most preferred. In the Multimodal learning styles preferences, it was found that 4% of the participants preferred a combination of visual and kinaesthetic learning styles. Interestingly, there was no significant relationship between learning styles and academic achievement in this study. Kamrun and colleagues (2022) investigated the relationship between academic performance and the learning styles and critical thinking of 469 medical science students in Iran. The instruments used were the Kolb’s learning styles and California’s CT skills test, and cumulative GPAs were used as the outcomes of academic performance. The results revealed that there was a significant relationship between academic performance and learning styles. Additionally, learning styles changed over time “among medicine and dentistry students from an abstract-reflexive style to a concrete-active one”. Hamed and Almabruk [18] examined the relationship between learning styles and the academic achievement of fourth-year English-major students in Libya. Kinsella’s [27] learning style questionnaire was employed to collect data. The results revealed that the tactile style was the dominant learning style, followed by the auditory style, and the kinaesthetic style was the least preferred learning style. There was a significant relationship between learning styles and academic achievement, and the strongest association was found between the auditory learning style and academic achievement. Karatus and Yalin [26] investigated the relationship between learning-teaching styles and students’ academic achievement. The Grasha-Reichmann Learning styles and the Teaching Styles Inventories were used to determine the students’ dominant learning styles and the instructors’ dominant teaching styles. The findings revealed that there was no significant difference between the students’ achievement scores and learning styles, suggesting that the achievement score did not change significantly in accord with their learning styles.

There have also been a few research studies focusing on elementary school students’ learning styles. For example, Sarican [55] explored the relationship between learning styles and the academic success of 163 G4 students in Istanbul, Turkey. The Marmara Learning Style Scale [58] was used to collect the data. Regression analysis and the content analysis of the qualitative data confirmed that learning styles were a predictor of academic achievement. Furthermore, Pürbudak and Usta [49] examined the relationship between the learning styles on the Web 2.0 environment and the academic achievement of 83 G6 students in Turkey, divided into the experimental group (*n* = 43) and the control group (*n* = 40). Kolb’s learning styles, computer thinking skill levels, online cooperative learning attitude scales, and an academic achievement test were applied to both the experimental and control groups as a pre-test and post-test. The results revealed that there was a meaningful difference between students’ academic achievement scores, online cooperative attitude levels, computer thinking skill levels, and learning styles. Additionally, the students with converging learning styles had higher level academic achievement, online cooperative level, and computer thinking skill level than the students with accommodating and diverging learning styles.

## The Big Five, learning styles, and academic success

The Big Five personality traits and learning styles of students have been widely investigated; unfortunately, not many research studies have focused on the relationships of both the Big Five and learning styles and students’ academic success. However, most of these studies suggested that both the Big Five personality traits and learning styles contributed to university students’ academic performance [30, 32, 36]. In investigating business administration and communication arts students’ personality types and learning styles, Pornsakulvanich and colleagues [47] found that personality traits were better predictors of academic performance than learning styles were. Conscientiousness, openness, and agreeableness were significant predictors of cognitive academic performance, whereas conscientiousness, openness, agreeableness, and emotional stability were significant predictors of affective academic performance. Learning styles were also significant predictors of cognitive academic performance. Additionally, in examining university students enrolled in e-learning courses. Siddiquei and Khalid [57] found that GPA was positively correlated with three personality traits (openness, agreeableness, and conscientiousness) and was negatively correlated with neuroticism. GPA was also positively correlated with the three learning styles: active, intuitive, and global.

**Fig. 1:**
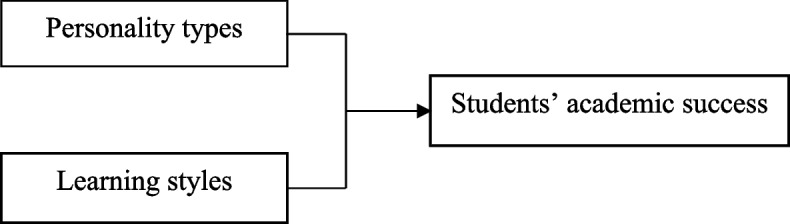
The general framework

Based on the literature review, it suggests that personality types are related to learning styles and both of them have a relationship with students’ academic success. Thus, the general framework for this study (Fig. 1) can be displayed as follows:

Additionally, in order to thoroughly explore the relationship between the two characteristics and students’ academic success, the framework has been further modified to investigate two more variables: school and gender.

## Conceptual association model

**Fig. 2:**
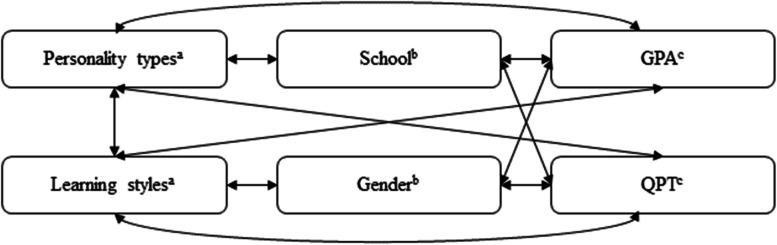
Relationships among personality types, learning styles, school, gender, GPA (students’ grade point average), and QPT (English proficiency score). ^a^ Independent variables. ^b^ Confounding variables. ^c^ Dependent variables

As shown in Fig. 2, in addition to the two independent variables, personality types and learning styles, two confounding variables (school and gender) have been added to the framework of the study.

## Research questions

Although there have been quite a few research studies on the relationships among the Big Five personality traits, learning styles, and academic performance, none of them has explored the use of the Learning Styles Indicator (LSI) scales as a data collection instrument [1, 12, 24, 26, 30, 32, 34, 43, 47, 49, 55, 57, 62]. This study found the LSI scales interesting, more relevant, and in accordance with active learning activities. We thus aimed to use the LSI scales and the IPIP to assess G12 students’ learning styles and personality types to collect and analyse the data to answer the following research questions:

1. What are the personality types of the participants?
2. What are the learning styles of the participants?
3. What is the relationship between personality types and learning styles?
4. What is the relationship between the participants’ personality types and their GPAs?
5. What is the relationship between the participants’ personality types and their English proficiency scores?
6. What is the relationship between the participants’ learning styles and their GPAs?
7. What is the relationship between the participants’ learning styles and their English proficiency scores?

## Methods

### Participants

The participants in the study were a total of 1,358 Grade-12 students from three secondary schools, in the first semester of academic year of 2022. Purposive sampling was employed to select the three largest government secondary schools, with one school chosen from each of the three districts in the northernmost province of Thailand to ensure geographic diversity. These students represented all academic programs offered: Science-Math (*N* = 503; 37.0%), Math-English (*N* = 130; 9.6%), others (*N* = 473; 34.8%), and missing (*N* = 252; 18.6%). They comprised 438 males (32.3%), 663 females (48.8%), and 257 missing (18.9%). (See Table 2.)

**Table 2:** Participants’ demographic information

Description		N	%
School	School A	373	27.5
	School B	448	33.0
	School C	537	39.5
	Total	1,358	100.0
Academic Program	Science-Math	503	37.0
	Math-English	130	9.6
	Others (Social studies, Languages, Vocational studies)	473	34.8
	Missing	252	18.6
	Total	1,358	100.0
Gender	Male	438	32.3
	Female	663	48.8
	Missing	257	18.9
	Total	1,358	100.0

## Instruments

### The International Personality Item Pool Scales (IPIP)

Goldberg’s [14] IPIP Big Five personality markers was adopted to assess the students’ personality traits. The 50-item version, where each personality trait has 10 items, was used. Example items include “I am the life of the party”, measuring extraversion, and “I pay attention to details”, measuring conscientiousness. A five-point Likert scale was used, ranging from “1” (strongly disagree) to “5” (strongly agree). The internal reliabilities or the coefficient alphas of the scales were as follows: 0.87 for Extroversion, 0.82 for Agreeableness, 0.79 for Conscientiousness, 0.86 for Neuroticism, 0.84 for Openness, and 0.84 for the whole IPIP scales. In order to ensure the understanding of the respondents, a Thai version of the IPIP scales was used. The original IPIP scales were translated into Thai adapting the double back-translation technique [25, 61, 64]. All of the items and instructions of the IPIP scales were first translated into Thai by a professional translator, and then back-translated into English by two translators independently. Subsequently, the three translators and the first author discussed online to validate the accuracy and appropriateness of the Thai-version IPIP scales.

## Learning styles indicator scales

The Learning Styles Indicator (LSI) [6, 63] was used to identify the students’ learning styles. The LSI scales were developed based on Reid’s [51] PLSPQ, an instrument used to assess L2 students’ learning styles. The LSI scales include three learning style scales: (1) Group activity orientation (GAO, 5 items), (2) Individual activity orientation (IAO, 7 items), and (3) Project orientation (PO, 11 items). Example items are: “I learn more when I study with a group” (GAO), “When I work alone, I learn better” (IAO), and “I learn more when I can make something for a class project” (PO). The LSI scales have been validated by using exploratory factor analysis and a triangular approach employing a questionnaire, semi-structured interviews, and participant observations [6]. The original LSI scales were translated into Thai following the same procedure of the double back-translation technique as previously explained in the development of the Thai-version IPIP scales.

## Academic achievement (GPA)

Students’ GPAs were collected via the registrar’s office of each school. In all schools, grades ranged from zero to four points, with higher values indicating better grades. The GPA is a general measure of academic achievement at the end of each semester. The GPAs used for the study were collected from the records of the students’ cumulative GPAs of the second semester of academic year 2021, as they were their most current cumulative GPAs.

## English proficiency (QPT)

The Quick Placement Test (QPT) was adopted to measure the students’ English proficiency. The 60-item QPT version 1, a paper and pen version, was used. The QPT is an English proficiency test developed by the university of Cambridge Local Examination Syndicate [45]. Prominent features of the QPT’s paper and pen version include: (1) assessment of reading, vocabulary, and grammar; (2) ease of use; (3) multiple choice format; and (4) concordance with the ALTE (Association of Language Testers in Europe) level and the CEFR (Common European Framework of Reference for Languages) level, as shown in Table 3.

**Table 3:** Concordance chart of equivalent levels

Paper and pen test score	ALTE Level	CEFR Level
0–17	0 Beginner	A1
18–29	1 Elementary	A2
30–39	2 Lower Intermediate	B1
40–47	Upper Intermediate	B2
48–54	Advanced	C1
55–60	Very Advanced	C2

The QPT is a standardized and widely recognized tool for measuring general English proficiency levels and is freely available for academic and placement purposes.

## Data collection

All of the data were collected from 1,358 Grade-12 students in three secondary schools, in the first semester of academic year 2022. The IPIP scales, the LSI scales, and the QPT were administered to students in each school with collaboration of their teachers to help proctor at the same time during a homeroom period. The entire process of the data collection in each school lasted 60 min. The data collection took place in each school during the first three weeks of August, 2022. The first author and a research assistant were present at each school during the data collection time to help the teachers. Additionally, students’ academic achievement GPA scores were collected via the registrar’s office of each school.

## Data analysis

Descriptive statistics were used to analyse the general demographic data of the participants. All of the completed questionnaire sheets were manually examined, and then analysed to identify each participant’s personality type and learning style in order to answer RQ 1 and RQ 2. A Chi-square test was used to determine whether there was a relationship between personality types and learning styles (RQ 3). For the other relationship research questions (RQ 4 through RQ 7), a multivariate analysis of variance (MANOVA) was performed. The statistical technique MANOVA was chosen because the researchers investigated two dependent variables that were continuous (GPA and QPT scores), whereas the independent variables and the confounding variables, gender and school were all categorial. The benefit of the multivariate approach over the univariate approach in this situation is that we were able to take into account the fact that there were two continuous dependent variables for each observation. Treating it as two separate independent univariate studies would thus leave out this additional information.

## Results

The data were collected from three different schools with the total number of 1,358 observations. A total of 1,088 students filled out the learning styles survey questionnaire and 1,109 students filled out the questionnaire on personality types. The dependent variables were GPA and QPT, and the independent variables were learning styles, personality types, and the latent variables used in the model were gender and school. The results of the study are presented in response to the seven research questions posed.

1. What are the personality types of the participants?

Table 4 on personality types reveals that the highest percent was personality type A (Agreeableness) at 59.7%, followed by C (Conscientiousness) at 12.2%, N (Neuroticism) at 10.3%, Ties (Multimodal) at 10.2%, O (Openness) at 4.8%, and finally E (Extraversion) at 2.9%.

**Table 4:** Personality types of the participants

		Frequency	Percent	Valid Percent	Cumulative Percent
Valid	A	662	48.7	59.7	59.7
	C	135	9.9	12.2	71.9
	E	32	2.4	2.9	74.8
	N	114	8.4	10.3	85.0
	O	53	3.9	4.8	89.8
	Tie	113	8.3	10.2	100.0
	Total	1109	81.7	100.0	
Missing		249	18.3		
Total		1358	100.0

RQ2: What are the learning styles of the participants?

 

Table 5 on learning styles reveals that the students’ preferred learning style was GAO (Group activity orientation) with approximately 40.3%, followed by PO (Project orientation) at 29.3%, and finally IAO (Individual activity orientation) at 23.9%. A total of 6.5% had ties (Multimodal) in learning styles.

**Table 5:** Learning styles of the participants

		Frequency	Percent	Valid Percent	Cumulative Percent
Valid	GAO	438	32.3	40.3	40.3
	IAO	260	19.1	23.9	64.2
	PO	319	23.5	29.3	93.5
	Ties	71	5.2	6.5	100.0
	Total	1088	80.1	100.0	
Missing		270	19.9		
Total		1358	100.0

RQ3: What is the relationship between personality types and learning styles?

 

The Chi-square test was used to determine if personality types and learning styles were independent or not. This test was used because both personality types and learning styles were categorical variables. The results in Table 6 determined that they were not independent and it was a statistically significant. Thus, the authors rejected the null hypothesis that personality types and learning styles are independent at the 0.01 level. The information in Table 7 reveals that GAO was the preferred learning styles of all personality types (A, *n* = 283; C, *n* = 46; E, *n* = 13; N, *n* = 39; O, *n* = 16; Ties (Multimodal), *n* = 41), with a total number of 438.

**Table 6:** Cross table of personality types and learning styles

		Personality Types						Total
		A	C	E	N	O	Tie	
Learning Styles	GAO	283	46	13	39	16	41	438
	IAO	140	42	8	27	10	33	260
	PO	212	38	9	20	15	25	319
	Ties	26	9	2	9	11	14	71
Total		661	135	32	95	52	113	1088

**Table 7:** The Chi-square test result

Chi-Square Test			
	Value	df	Asymp. Sig. (2-sided)
Pearson Chi-Square	48.68^a^	15	.000
Likelihood Ratio	42.29	15	.000
N of Valid Cases	1088		

a. 2 cells (8.3%) have an expected count less than 5. The minimum expected count is 2.09

As personality type A was the main personality types, comprising 59.7% of the participants (see Table 4), it was the dominant group of all learning styles: GAO (*n* = 283), PO (*n* = 212), IAO (*n* = 140), and ties or Multimodal (*n* = 26). In like manner, personality type E (the smallest group, *n* = 32) turned out to be a very small group in each of the learning styles, as shown in Table 7.

RQ4: What is the relationship between the participants’ personality types and their GPAs?
RQ5: What is the relationship between the participants’ personality types and their English proficiency scores?
RQ6: What is the relationship between the participants’ learning styles and their GPAs?
RQ7: What is the relationship between the participants’ learning styles and their English proficiency scores?

In order to answer RQ4 through RQ7, a multivariate analysis of variance (MANOVA) was performed (see Table 8). The statistical technique MANOVA was used because the investigation involved two dependent variables that were continuous, GPA and QPT (English proficiency scores), whereas the independent variables, including the confounding variables, school and gender, were all categorical. The multivariate approach is able to take into account the fact that there are two continuous dependent variables for each observation. Treating it as two separate independent univariate studies would leave out this additional information.

**Table 8:** MANOVA results

Multivariate Tests^a^						
Effect		Value	F	Hypothesis df	Error df	Sig.
Intercept	Pillai’s Trace	.97	15,959.02^b^	2	1052	0.000
	Wilks’ Lambda	.03	15,959.02^b^	2	1052	0.000
	Hotelling’s Trace	30.34	15,959.02^b^	2	1052	0.000
	Roy’s Largest Root	30.34	15,959.02^b^	2	1052	0.000
School	Pillai’s Trace	.37	119.62	4	2106	.000
	Wilks’ Lambda	.63	136.79^b^	4	2104	.000
	Hotelling’s Trace	.59	154.39	4	2102	.000
	Roy’s Largest Root	.59	309.21^c^	2	1053	.000
Gender	Pillai’s Trace	.06	33.86^b^	2	1052	.000
	Wilks’ Lambda	.94	33.86^b^	2	1052	.000
	Hotelling’s Trace	.06	33.86^b^	2	1052	.000
	Roy’s Largest Root	.06	33.86^b^	2	1052	.000
Learning Styles	Pillai’s Trace	.02	3.68	6	2106	.001
	Wilks’ Lambda	.98	3.70^b^	6	2104	.001
	Hotelling’s Trace	.02	3.71	6	2102	.001
	Roy’s Largest Root	.02	7.38^c^	3	1053	.000
Personality Types	Pillai’s Trace	.02	1.62	10	2106	.094
	Wilks’ Lambda	.99	1.62\^b^	10	2104	.095
	Hotelling’s Trace	.02	1.62	10	2102	.095
	Roy’s Largest Root	.01	1.86^c^	5	1053	.098

a. Design: Intercept + School + genderno3 + Learning Styles + Personality Types

b. Exact statistic

c. The statistic is an upper bound on F that yields a lower bound on the significance level

On the multivariate level, that is when considering GPA and English proficiency (QPT scores) at the same time, it was found that school, gender, and learning styles were all significant at the 0.01 level and the personality types were significant only at the 0.10 level. That is, taking account of gender and school, learning style was significant at the 0.01 level, confirming that there was a relationship between learning styles and GPA and the QPT scores (RQ 6 and RQ7).

Unfortunately, taking into account the gender and school, personality types were significant at the 0.10 level but not at the 0.05 level. Thus, it did not confirm a relationship between personality types and GPA or the QPT scores, for RQ4 and RQ5 at the 0.05 level but only at the 0.10 level. It should be noted that personality types were related to learning styles, so in a sense there was an overlap in information and including both personality types and learning styles in the model has lessened the importance of personality types.

On the univariate level two ANOVAs breaking out QPT and GPA (see Table 9), it was found that school was significant at the 0.01 level on both QPT and GPA, and gender was only significant in terms of GPA. Learning styles were significant at the 0.01 level for GPA and at the 0.05 level for QPT, confirming RQ6 and RQ7, respectively. Personality types were significant at the 0.10 level for both GPA and QPT; thus, personality types were not significant on the univariate level and did not confirm RQ4 or RQ5.

**Table 9:** ANOVA results

Tests of Between-Subjects Effects						
Source		Type III Sum of Squares	df	Mean Square	F	Sig
Corrected Model	QPT	14,993.04^a^	11	1363.00	44.09	.000
	GPA	59.58^b^	11	5.42	36.09	.000
Intercept	QPT	183,108.71	1	183,108.71	5923.15	.000
	GPA	4360.39	1	4360.39	29,050.35	.000
School	QPT	13,392.91	2	6696.46	216.62	.000
	GPA	40.24	2	20.12	134.04	.000
Gender	QPT	7.37	1	7.37	.24	.625
	GPA	9.79	1	9.78	65.15	.000
Learning Styles	QPT	346.76	3	115.59	3.74	.011
	GPA	2.12	3	.71	4.71	.003
Personality Types	QPT	274.57	5	54.92	1.78	.115
	GPA	1.07	5	.21	1.42	.213
Error	QPT	32,552.52	1053	30.91		
	GPA	158.05	1053	.15		
Total	QPT	599,808.00	1065			
	GPA	13,250.78	1065			
Corrected Total	QPT	47,545.56	1064			
	GPA	217.64	1064			

a. R Squared =.315 (Adjusted R Squared =.308)

b. R Squared =.274 (Adjusted R Squared =.266)

## Discussion and conclusion

The present study aimed to investigate the relationships among the Big Five personality traits, learning styles, academic achievement, and English proficiency of Grade 12 EFL students. Seven research questions guided the inquiry, and the findings yielded valuable insights into how these psychological and pedagogical variables interact in a secondary education context.

In terms of personality distribution (RQ1), the results revealed that Agreeableness was the most dominant personality trait among participants (59.7%), followed by Conscientiousness (12.2%), Neuroticism (10.3%), Multimodal (10.2%), Openness (4.8%), and Extraversion (2.9%). These findings echo previous research by Pornsakulvanich et al. [47], who found that Thai university students also scored highest on agreeableness and conscientiousness. Komin’s [31] framework further explains these tendencies, suggesting that Thai individuals tend to emphasize harmonious, pleasant, and cooperative interpersonal relationships. Traits commonly associated with agreeableness, such as kindness, sympathy, and cooperation [13], appear to be prevalent among Thai secondary school students as well.

As for students’ learning preferences (RQ2), the study found that Group Activity Orientation (GAO) was the most favored learning style (40.3%), followed by Project Orientation (PO) (29.3%), Individual Activity Orientation (IAO) (23.9%), and Multimodal (6.5%). These results indicate that most students preferred collaborative, experiential, and project-based learning approaches. A smaller group preferred working individually, which may reflect traits of self-reliance or introversion. These findings suggest that learning environments that promote teamwork and active engagement may be more suitable for the majority of Thai EFL learners at the secondary level.

Regarding the relationship between personality traits and learning styles (RQ3), a chi-square test revealed that the two variables were significantly associated. A majority of students across all learning style categories, GAO, PO, IAO, and Multimodal, were of the agreeableness type. This relationship supports the idea that students with similar personality profiles may also exhibit shared preferences in how they approach learning tasks and classroom engagement.

Research Questions 4 through 7 focused on the relationships among personality traits, learning styles, academic achievement (GPA), and English proficiency scores (QPT). A MANOVA revealed significant relationships among school, gender, and learning styles (*p* < 0.01), while personality traits showed marginal significance (*p* < 0.10). Further univariate analysis indicated that school significantly affected both QPT and GPA (*p* < 0.01), while gender was only significant for GPA. Learning styles were significantly associated with QPT (*p* < 0.05) and GPA (*p* < 0.01), while personality traits did not reach the conventional significance level (*p* < 0.05), showing only marginal significance. These findings align with studies such as Komarraju et al. [30], Köseoglu [32], and Marcela [36], but contrast with Pornsakulvanich et al. [47], who found personality traits to be stronger predictors of academic performance than learning styles. This discrepancy may stem from differences in the populations studied, as most prior research focused on university students, whereas this study examined secondary school students. The developmental stage of secondary school students may moderate how personality traits influence academic outcomes, warranting further exploration.

The study offers both academic and practical contributions. Academically, it extends current knowledge by examining the interplay between personality and learning styles in an underexplored population, Grade 12 Thai EFL learners. The study also implemented an activity-oriented learning style model comprising GAO, IAO, and PO, offering a framework for categorizing learners beyond traditional cognitive or sensory preferences. Furthermore, the modified Learning Style Inventory (LSI) based on Reid’s [51] PLSPQ was adapted for L2 learners, representing a novel approach to assessing learning styles in the EFL context.

Practically, the findings underscore the importance of recognizing individual differences in the classroom. Educators and policymakers should acknowledge that students with different personality traits and learning preferences benefit from varied instructional methods and classroom activities. Providing opportunities for both collaborative and individual learning can help improve academic achievement and engagement. Moreover, adopting flexible teaching strategies can prepare students for diverse real-world scenarios where adaptability is essential [9, 12]. By understanding the interplay between personality and learning styles, educators can better support students’ English proficiency and overall academic success.

Despite these contributions, the study is not without limitations. First, the relationships among individual personality traits, learning styles, and academic performance could be explored in greater detail. Second, since differences were observed between male and female students in terms of learning style preferences, future research should investigate these gender-based differences more closely. Third, the instruments used, IPIP and LSI, may carry cultural biases in translation; validation in Thai contexts is recommended. Fourth, longitudinal research would be beneficial to examine how learning styles evolve over time. Lastly, employing mixed methods could help overcome the limitations of a purely quantitative design and provide richer insights into how personality and learning styles influence academic outcomes.

## Data Availability

No datasets were generated or analysed during the current study.
